# A heterogeneous phantom study for investigating the stability of PET images radiomic features with varying reconstruction settings

**DOI:** 10.3389/fnume.2023.1078536

**Published:** 2023-02-14

**Authors:** Emad Alsyed, Rhodri Smith, Lee Bartley, Christopher Marshall, Emiliano Spezi

**Affiliations:** ^1^ School of Engineering, Cardiff University, Cardiff, United Kingdom; ^2^ Wales Research and Diagnostic Positron Emission Tomography Imaging Centre (PETIC), Cardiff University, Cardiff, United Kingdom; ^3^ Department of Nuclear Engineering, Faculty of Engineering, King Abdulaziz University, Jeddah, Saudi Arabia

**Keywords:** radiomics, PET, cancer, reconstruction settings, stability analysis, physical phantoms

## Abstract

The purpose of this work was to assess the capability of radiomic features in distinguishing PET image regions with different uptake patterns. Furthermore, we assessed the stability of PET radiomic features with varying image reconstruction settings. An in-house phantom was designed and constructed, consisting of homogenous and heterogenous artificial phantom inserts. Four artificially constructed inserts were placed into a water filled phantom and filled with varying levels of radioactivity to simulate homogeneous and heterogeneous uptake patterns. The phantom was imaged for 80 min. PET images were reconstructed whilst varying reconstruction parameters. The parameters adjusted included, number of ordered subsets, number of iterations, use of time-of-flight and filter cut off. Regions of interest (ROI) were established by segmentation of the phantom inserts from the reconstructed images. In total seventy eight 3D radiomic features for each ROI with unique reconstructed parameters were extracted. The Friedman test was used to determine the statistical power of each radiomic feature in differentiating phantom inserts with different hetero/homogeneous configurations. The Coefficient of Variation (COV) of each feature, with respect to the reconstruction setting was used to determine feature stability. Forty three out of seventy eight radiomic features were found to be stable (COV ≤5%) against all reconstruction settings. To provide any utility, stable features are required to differentiate between regions with different hetro/homogeneity. Of the forty three stable features, fifteen (35%) features showed a statistically significant difference between the artificially constructed inserts. Such features included GLCM (Difference average, Difference entropy, Dissimilarity and Inverse difference), GLRL (Long run emphasis, Grey level non uniformity and Run percentage) and NGTDM (Complexity and Strength). The finding of this work suggests that radiomic features are capable of distinguishing between radioactive distribution patterns that demonstrate different levels of heterogeneity. Therefore, radiomic features could serve as an adjuvant diagnostic tool along with traditional imaging. However, the choice of the radiomic features needs to account for variability introduced when different reconstruction settings are used. Standardization of PET image reconstruction settings across sites performing radiomic analysis in multi-centre trials should be considered.

## 1. Introduction

Medical imaging modalities such as Positron Emission Tomography (PET), Computed Tomography (CT) and Magnetic Resonance (MR) contribute significantly in all phases of cancer management (1). PET imaging plays a fundamental role in qualitative assessment of several types of cancer (2). PET images are more often assessed visually by radiologists and clinicians (3). However, PET images traditionally provide a limited number of quantitative parameters such as the maximum, minimum and peak standardized uptake value (SUVmax, SUVmean, SUVpeak) (3, 4). These parameters are commonly used for quantifying tumor characteristics. SUVmax, for example, can be used to detect occult metastatic nodes in oral cancers (5). Additional quantitative parameters in the form of texture features have been proposed and are a current research topic in quantitative PET imaging. These have the potential to improve prognosis and diagnosis of patients with cancer (6). The past few years have seen increasingly rapid advances in the field of tumor textural analysis. Radiomics may be defined as a method of the extraction of quantitative imaging textures or features that cannot be seen by the human eye (7, 8). A considerable amount of literature has been published on the use of radiomic features. For example the utility of radiomic features as predictors of patient outcome and treatment response (9, 10).

The use of radiomic features as metrics in prognosis and diagnosis for several cancers is a promising development. However, with different imaging equipment, acquisition protocols and image processing, the variation and accuracy of radiomic features remains problematic and serves as a challenge to implementing radiomic features as biomarkers (11). There have been several investigations into the effect of different variables on the stability of PET images radiomic features. The impact of PET image reconstruction settings has been investigated (12–15). Several attempts have been made to investigate the impact of other conditions including factors such as respiratory motion (16), segmentation (17) and interpolation (18)) all of which may confound the utility of PET radiomic features. Pfaehler et al. (19) investigated the impact of different variables such as image reconstruction settings, noise, discretization method, and delineation method on the repeatability of 18F-FDG PET radiomic features. In a study which set out to determine the impact of reconstruction settings on 61 texture and features, Yan et al. (20) found that variation occurred when different reconstruction settings were applied. In their study, cluster shade, and zone percentage exhibited large variations. Features such as difference entropy, inverse difference normalized, inverse difference moment normalized, low gray level run emphasis, high gray level run emphasis, and low gray level zone emphasis were found to have high stability.

Clinical studies are complex and influenced by several factors including patient physiology and organ motion. For this reason, phantom studies can be a reasonable substitute to control for bias relative to biological variability of clinical studies. Previous research involving phantom experiments have mostly dealt with homogenous phantom images, and studies that analyse heterogeneous phantom images are limited (21–23). This study therefore set out to assess not only the effect of reconstruction settings on the stability of PET radiomic features, but also the ability of the radiomic feature to distinguish between homogeneous and heterogeneous uptake patterns. For this purpose, we designed a heterogeneous PET phantom comprising of four artificially constructed tumor inserts. The phantom was scanned and images were reconstructed with different reconstruction settings including number of ordered subsets expectation maximization (OSEM) subsets, number of iterations, use of time-of-flight (TOF) and filter cut off.

## 2. Materials and methods

### 2.1. Preparation

**Figure 1:**
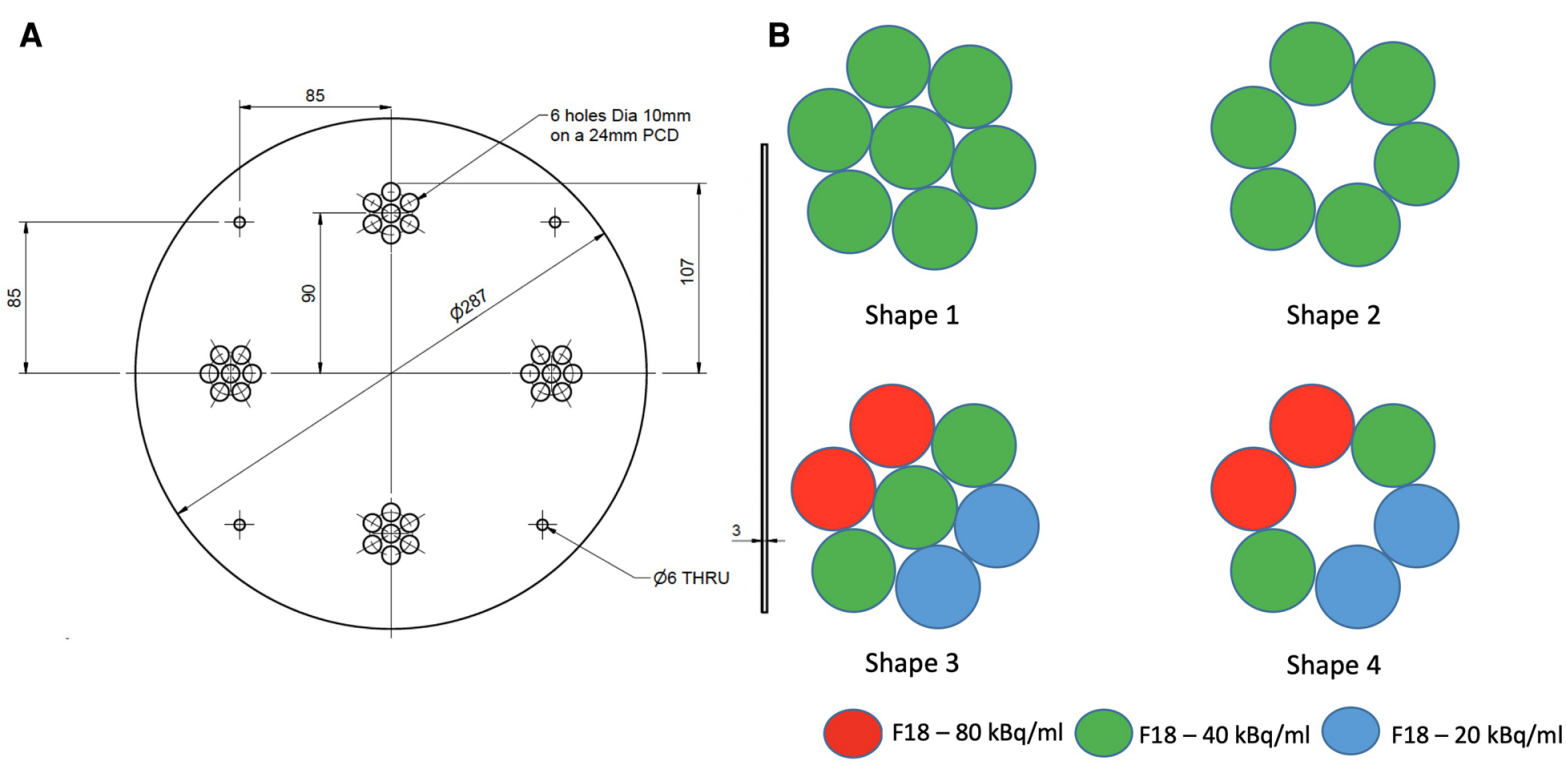
An illustrative layout of the syringe mounting plate (**A**) and four configurations of the artificial tumour inserts (**B**).

We designed a mounting plate made of PETG (polyester) capable of holding four artificially constructed inserts (38 mm PCD each) at 90 degrees to each other. Each insert consists of 7 syringes filled with different radioactivity concentrations to model lesions with varying degrees of heterogeneity. Two configurations of homogeneous tumour inserts were constructed by arranging 6 and 7 syringes, which were filled with 40 kBq/ml F-18 activity concentration to mimic tumors (≈145 cm^3^) with and without necrotic regions, respectively. The two remaining inserts were constructed in a similar way by arranging syringes filled with 3 different F-18 activity concentrations (20, 40 and 80 kBq/ml) to mimic heterogeneous tumors with and without necrotic regions, respectively. The extremes of concentrations chosen were based on the ratio of the highest and lowest intensities observed in a subset (n=10) of randomly chosen tumors from oesophageal cancer PET images. The average ratio between the highest and lowest intensity in this subset was 4:1, hence 80 and 20 kBq/ml were chosen as the maximum and minimum radioactivity concentrations in the phantom. The constructed inserts were placed in a cylindrical uniform water (5 kBq/ml F-18) filled phantom. Figure 1 shows an illustrative layout of the four configurations of artificial constructed tumour inserts.

### 2.2. Acquisitions and Reconstructions

**Figure 2:**
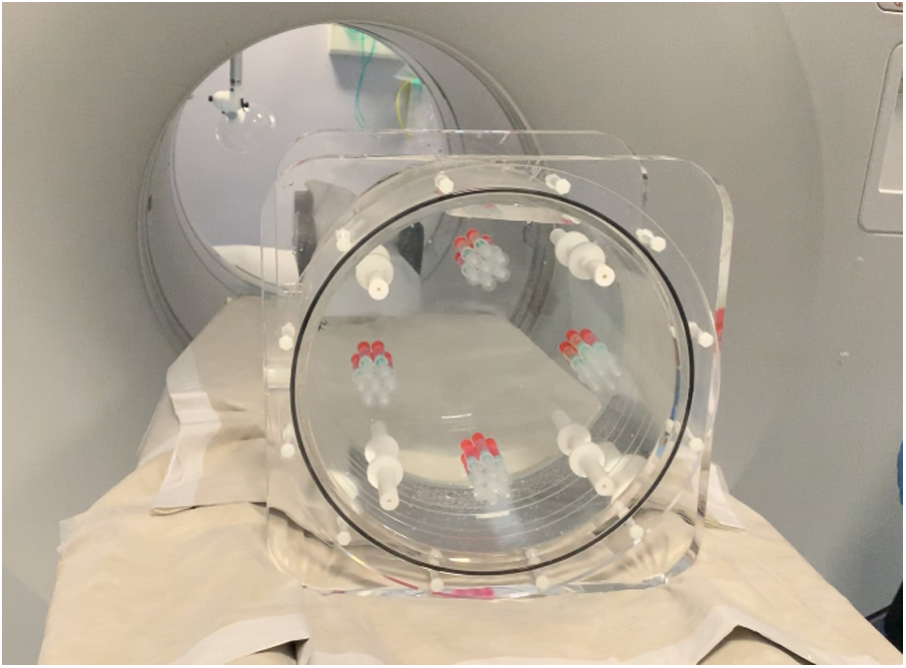
A picture of the designed phantom after placed on the scanner.

A GE Discovery 690 PET/CT scanner was used to acquire phantom images. Figure 2 shows a picture of the designed phantom placed on the scanner couch. The phantom was scanned for 80 min and images were reconstructed using the default settings that are used clinically (reference image): order subset expectation maximization (OSEM), point spread function (PSF) correction, Time-of-Flight (TOF) on, 24 subsets, 2 iterations, 6.4 mm filter cutoff and 256 matrix size. To evaluate the effect of reconstruction settings on image radiomic features, images were reconstructed with varying reconstruction settings including: number of subsets, number of iterations, filter cut-off and application of TOF. Table 1 shows the reconstruction parameters used to generate new images.

**Table 1:** List of reconstruction settings used to generate new images.

Reconstruction parameters	Variations
Number of subsets	12, 16, 18, 24, 32
Number of iterations	1, 2, 3, 4, 5, 6
Filter cut-off	0, 1, 2, 3, 4, 5, 6, 7
TOF	Yes, No

Default settings: TOF, 24 OSEM subsets, 2 iterations, 6.4 mm filter cutoff.

### 2.3. Segmentation

**Figure 3:**
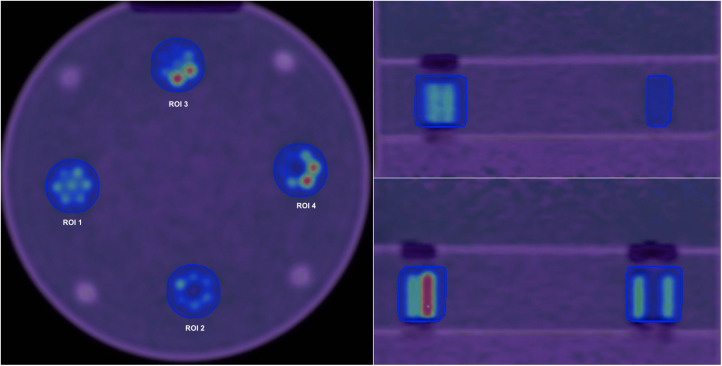
Axial (left), Coronal (right, top) and Sagittal (right, bottom) views for the phantom scan at 80 min and default reconstruction settings. Four different regions of interest are shown in the axial view.

Velocity 3.2.1 software (Varian Medical Systems, Atlanta, USA) was used to obtain the ground truth contour from the first configuration (homogeneous tumour). To remove variability in ROI delineation, this contour was overlaid onto all other configurations and other subsequent images resulting from images reconstructed with different reconstruction settings. Figure 3 shows Axial, Coronal and Sagittal views for the phantom scan at 80 min.

### 2.4. Features extraction and data analysis

For each region of interest (ROI), SPAARC (Spaarc Pipeline for Automated Analysis and Radiomic Computing), an in-house developed tool built with Matlab, was used to extract 78 3D-radiomic features (18, 24). Features including a 25 gray level co-occurrence matrix (GLCM), 16 gray-level run-length matrix (GLRLM), 16 gray-level size zone matrix (GLSZM), 16 Gray-level distance zone matrix (GLDZM) and 5 neighborhood gray-tone difference matrix (NGTDM) were extracted. SPAARC radiomic analysis is standardized according to the Image Biomarker Standardization Initiative (IBSI) (25). All extracted features are listed in Table 2.

**Table 2:** List of extracted radiomic features.

Features group	Features	Features group	Features
GLCM	Joint maximum	GLSZM	Small zone emphasis
	Joint average		Large zone emphasis
	Joint variance		Low grey level zone emphasis
	Joint entropy		High grey level zone emphasis
	Difference average		Small zone low grey level emphasis
	Difference variance		Small zone high grey level emphasis
	Difference entropy		Large zone low grey level emphasis
	Sum average		Large zone high grey level emphasis
	Sum variance		Grey level non-uniformity
	Sum entropy		Grey level non-uniformity normalised
	Angular second moment		Zone size nonuniformity
	Contrast		Zone size non-uniformity normalised
	Dissimilarity		Zone percentage
	Inverse difference		Grey level variance
	Inverse difference normalised		Zone size variance
	Inverse difference moment		Zone size entropy
	Inverse difference moment normalised		
	Inverse variance		
	Correlation		
	Autocorrelation		
	Cluster tendency		
	Cluster shade		
	Cluster prominence		
	First measure of information correlation		
	Second measure of information correlation		
GLRLM	Short runs emphasis	GLDZM	Small distance emphasis
	Long runs emphasis		Large distance emphasis
	Low grey level run emphasis		Low grey level zone emphasis
	High grey level run emphasis		High grey level zone emphasis
	Short run low grey level emphasis		Small distance low grey level emphasis
	Short run high grey level emphasis		Small distance high grey level emphasis
	Long run low grey level emphasis		Large distance low grey level emphasis
	Long run high grey level emphasis		Large distance high grey level emphasis
	Grey level nonuniformity		Grey level non-uniformity
	Grey level non-uniformity normalised		Grey level non-uniformity normalised
	Run length non-uniformity		Zone distance non-uniformity
	Run length non-uniformity normalised		Zone distance non-uniformity normalised
	Run percentage		Zone percentage
	Grey level variance		Grey level variance
	Run length variance		Zone distance variance
	Run entropy		Zone distance entropy
NGTDM	Coarseness		
	Contrast		
	Busyness		
	Complexity		
	Strength		

To evaluate each feature's stability when extracted with different reconstruction settings, the coefficient of variation (COV) was calculated. COV serves as a simple measurement in evaluating the variability of feature measurements and is one of the most widely used methods in assessing the stability of radiomic features (14, 20, 22). COV is the ratio of the standard deviation to the mean and it can be expressed as the following equation:

$$\mathrm{COV}=\frac{\text{( Standard Deviation) }\times 100}{\text{Mean}}$$

In this study, we categorized features based on their COV values and established four groups; stable (COV≤5%), moderately stable (5%<COV≤10%), poorly stable (10%<COV≤20%) and unstable (COV>20%). The categorization approach taken in this study is based on Yan et al. (20) and Shiri et al. (14).

Features that demonstrated stability were analysed using the Friedman test (26), to determine if they were capable of discerning, with statistical significance, difference between phantom objects with varying heterogeneity. The Friedman test is a non-parametric test that determines the statistical significance of differences in dependent variables (texture features) between groups (homogeneity). The Friedman test involves ranking each row (features values in each reconstruction parameter) separately and then sums the ranks in each column (regions). In our study, rows contain feature values at different reconstruction settings. The p value will be small if the sums are very different. In contrast, high p values indicate that there is no significant difference between tested groups.

The Friedman test was performed for each feature to determine whether or not there is a statistically significant difference between the regions used in each configuration, whilst varying reconstruction parameter settings (shape1 vs shape2, shape1 vs shape3, shape1 vs shape4, shape2 vs shape3, shape2 vs shape4 and shape3 vs shape4). The steps of applying the Friedman test can be summarized as follows:

1) Ranking for each feature the values obtained from the varying reconstructions (row) in ascending order.
2) The sum of ranks for each region (column) was calculated.
3) The test statistic (Q) was calculated using the following equation:
  $$Q=\frac{12}{nk(k+1)}\sum_{\;j=1}^{k}R_{j}^{2}-3n(k+1)$$
  where: n, number of reconstruction parameters = 21; k, number of regions = 2 (each combination consists of 2 regions); $Rj^{2}$, sum of ranks for the jth region.
4) Determining corresponding p value.

**Figure 4:**
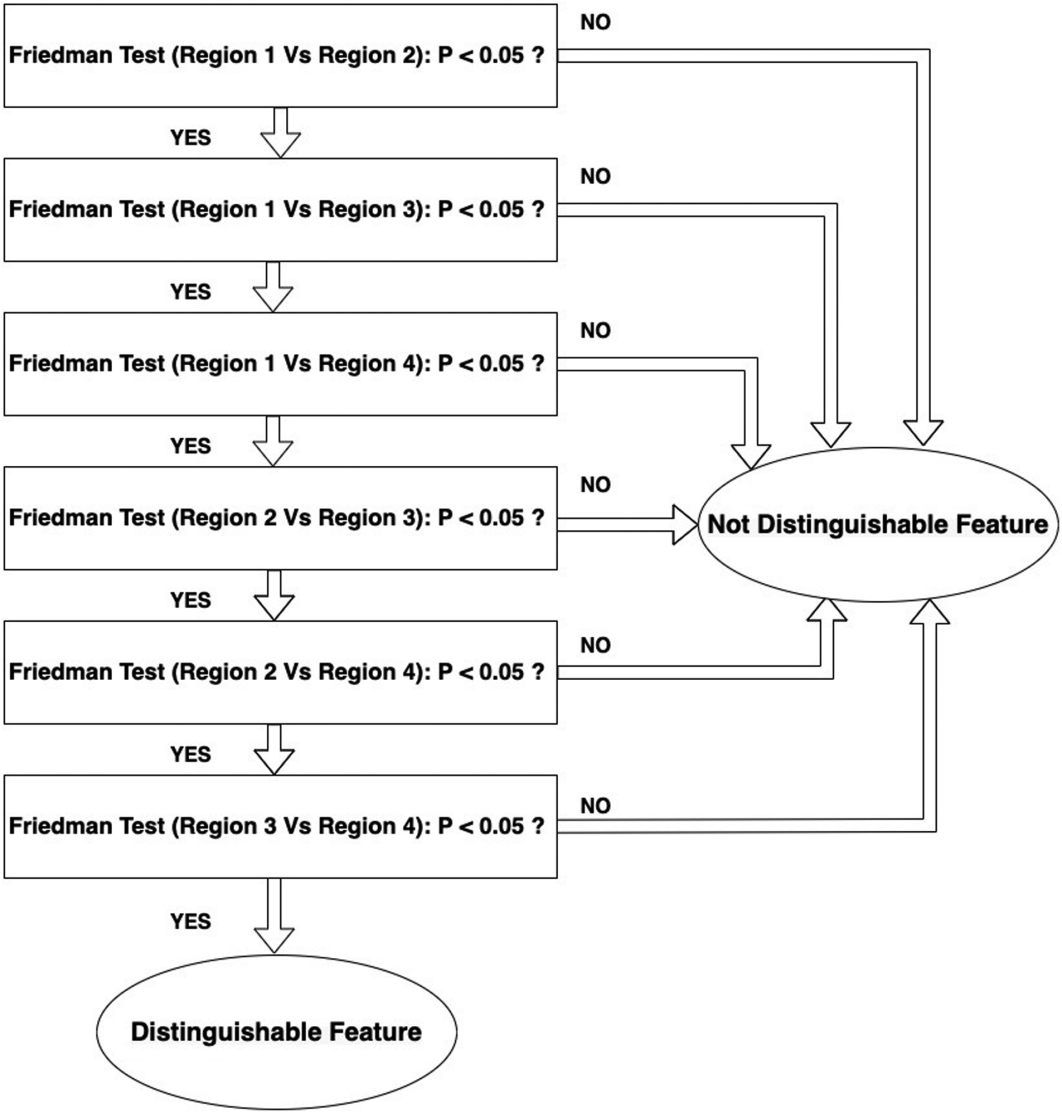
Workflow of selecting distinguishable features that can detect the differences between the regions.

**Figure 5:**
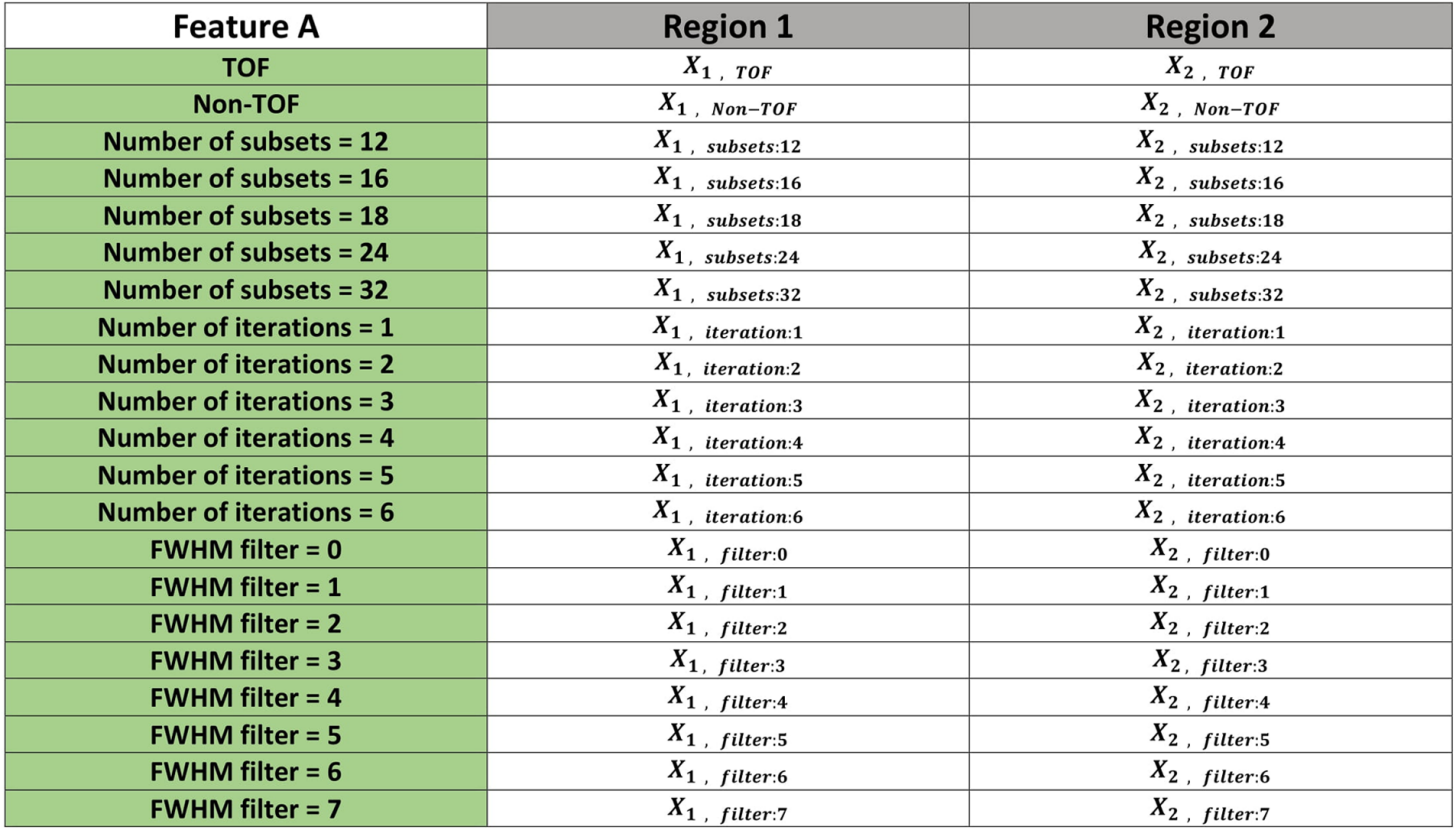
An illustrative example showing how the data sorted to perform the Friedman test. The example includes 21 reconstruction settings and two different regions (shape1 vs shape2). This was repeated for each of five other combinations (shape1 vs shape3, shape1 vs shape4, shape2 vs shape3, shape2 vs shape4 and shape3 vs shape4). P values were then calculated for each pair of regions to determine whether or not there is a statistically significant difference between the means of the regions.

The null hypothesis for the Friedman test is that there are no differences between dependent variables (texture features obtained with varying reconstruction parameters). If the calculated probability is low, (p less than the selected significance level) the null-hypothesis is rejected and we can assert that the texture feature allows separation between the paired regions. If the p value is higher than significance the null hypothesis is accepted and the texture parameter shows no difference between the paired regions. A significance level of 0.05 was chosen. If any feature demonstrated significance for all of the 6 paired combinations the feature will be considered as distinguishable feature to capture heterogeneity differences in the phantom. The workflow for this analysis is shown in Figure 4. Figure 5 illustrates how the data is sorted (in form of a table) to perform the Friedman test.

## 3. Results

**Figure 6:**
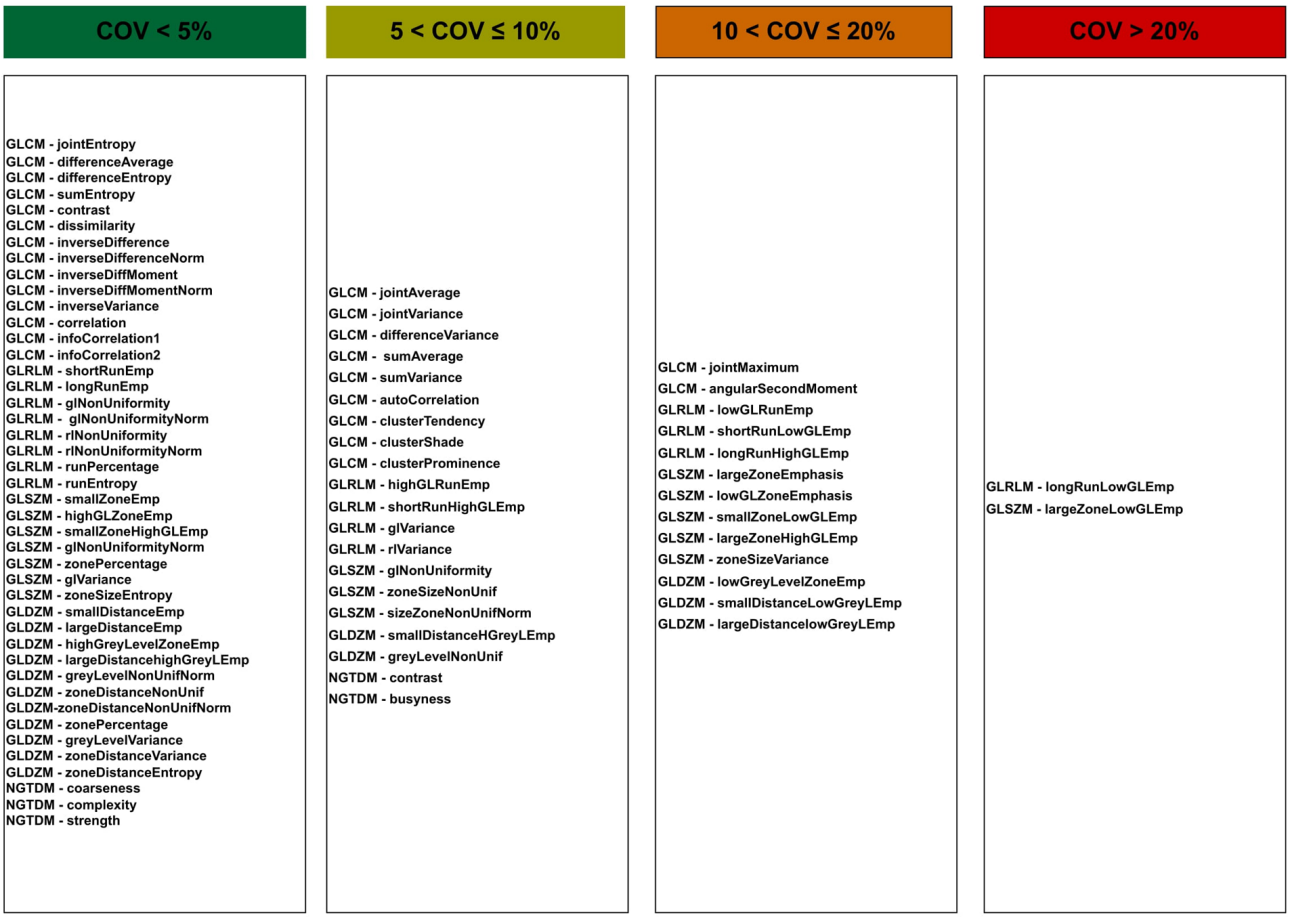
Features for each category of stability over all reconstruction settings.

Figure 6 indicates features as categorised based on the average of the COV over all tested reconstruction settings. Forty three features were found to be stable (COV≤5%) with the application of different reconstruction settings. Such stable features included GLCM (Difference entropy, Inverse difference normalised, Inverse difference moment normalised, Second measure of information correlation and 10 other features), GLRL (Short runs emphasis, Run percentage, Run entropy and 5 other features), GLSZM (Zone size entropy and 6 other features), GLDZM (Zone distance entropy and 10 other features), NGTDM (strength, coarseness, complexity).

Figure 6 also shows 20 and 13 features with moderate (5%<COV≤10%) and poor (5%<COV≤10%) stability over all reconstruction settings, respectively. Only two features show high variation (COV>20%). Unstable features included GLRLM (Long run low grey level emphasis) and GLSZM (Large zone low grey level emphasis).

**Figure 7:**
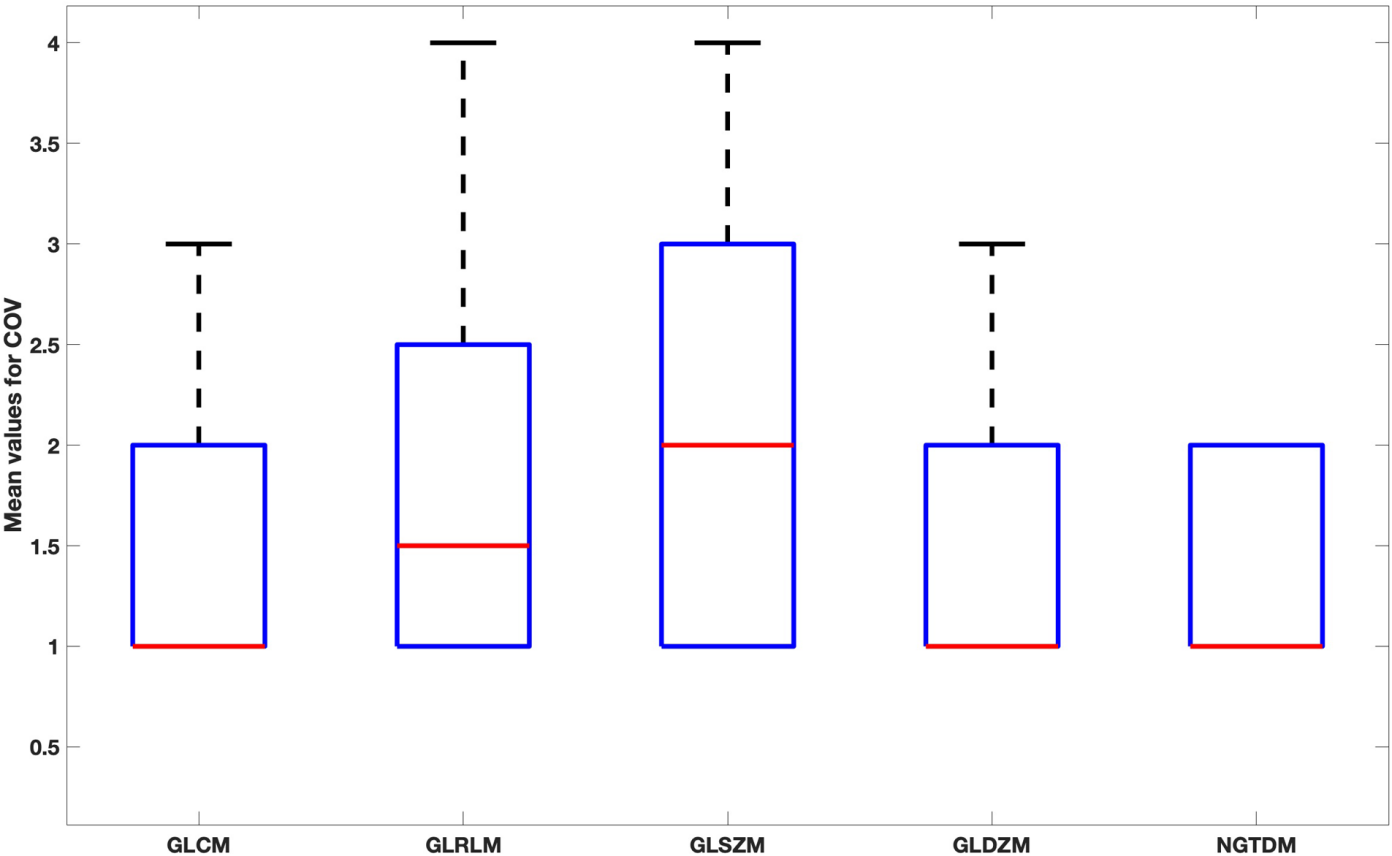
Box plot for the mean values of COV for each feature family over all reconstruction settings.

When comparing feature groups, NGTDM features have the lowest mean COV (Figure 7). GLSZM was the most sensitive feature type to the reconstruction settings.

### 3.1. Impact of TOF

**Figure 8:**
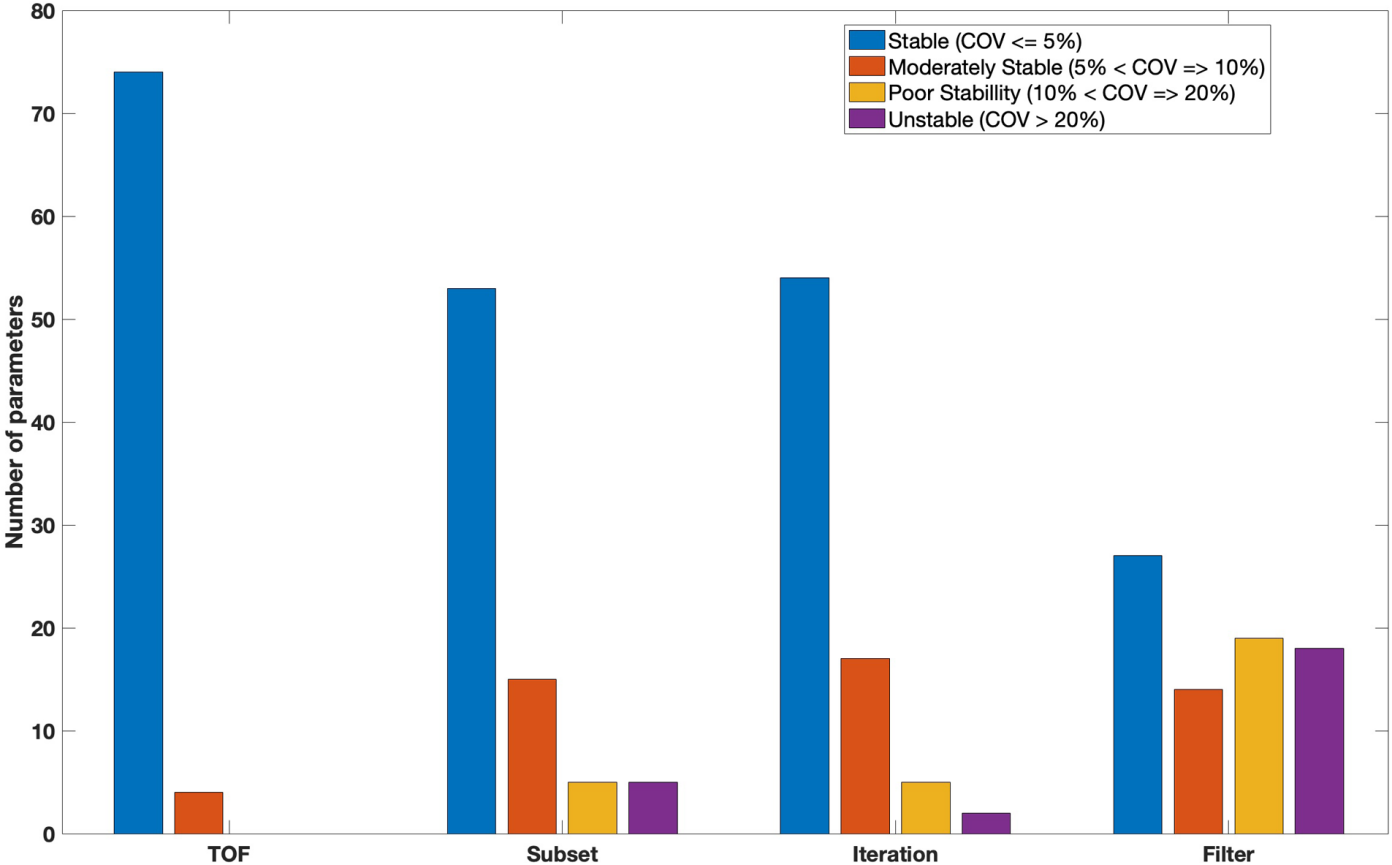
Bar chart showing the number of features for each category.

As shown in Figure 8, Seventy four features were stable against the use of TOF. Such stable features included GLCM (joint entropy, difference average,sum entropy, correlation, joint maximum), GLRLM (shortRunEmp, Long run high grey level emphasis, Grey level non uniformity, run percentage), GLSZM (Small zone emphasis, Zone percentage, Zone size entropy), GLDZM (Small distance emphasis, Large distance emphasis, Low grey level zone emphasis, Zone distance variance) and NGTDM (Coarseness, Busyness, Complexity, Strength). Only four features (GLCM-Contrast, GLSZM-Zone Size Variance, GLSZM-Large Zone Emphasis and NGTDM-Contrast) demonstrated moderate stability against TOF. No features were poorly stable or unstable.

### 3.2. Impact of number of subsets

Figure 8 also showed that fifty three features were classed as having high stability (COV≤5%) with varying number of OSEM subsets. Fifteen features (19%) were classed as having moderate stability (5%<COV≤10%). Five features including GLRL (Run length variance), GLSZM (Zone size non uniformity, Small zone low grey level emphasis) and GLDZM (Large distance low grey level emphasis, Small distance low grey level emphasis) were poorly stable. The remaining features (5) such as GLRLM (Low grey level run emphasis) and GLSZM (Large zone high grey level emphasis) had high variability (unstable) at different number of subsets. All features from NGTDM were stable (COV≤5%) with varying the number of subsets during reconstruction.

### 3.3. Impact of the number of iterations

More than 60% (54) of features were found to be stable with different number of iterations (Figure 8). Features with very low variation included GLCM (sum average, sum variance, sum entropy, contrast, dissimilarity, inverse difference, inverse difference normalised), GLRLM (run percentage, grey level Variance, run entropy), GLSZM (Small zone emphasis, High grey level zone emphasis , Small zone low grey level emphasis), GLDZM (Small distance high grey level emphasis, Large distance high grey level emphasis, Grey level non-uniformity normalised) and NGTDM (coarseness, busyness, complexity). Seventeen and five (GLRL-Low grey level run emphasis, GLRL-Short run low grey level emphasis, GLSZM-Small zone low grey level emphasis, GLSZM-Large zone high grey level emphasis, GLDZM-Large distance low grey level emphasis) features showed moderately stable and poorly stable against the number of iterations, respectively. Only two features GLRLM (Long run low grey level emphasis) and GLSZM (Large zone low grey level emphasis) showed large variation (COV>20%) with different numbers of iterations.

### 3.4. Impact of FWHM of the Gaussian filter

With changing FWHM of a Gaussian filter, twenty seven features showed very small variation (COV≤5%). 18% (14) and 24% (19) of features were found to be moderately stable and poorly stable, respectively. Eighteen features such as GLCM (cluster Shade, joint maximum, auto correlation), GLRLM (High grey level run emphasis , short run high grey level emphasis), GLSZM (large zone emphasis), GLDZM (Small distance low grey level emphasis) and NGTDM (busyness) demonstrated high variation (COV>20%) (Figure 8).

### 3.5. Analysis of Friedman test

Forty three features demonstrated high stability over all reconstruction settings. The Friedman test was used to find out how many of them differed statistically between regions. Fifteen out of 43 (35%) features showed statistically significant difference between regions and hence classed as distinguishable. Table 3 presents all of these distinguishable features. More than half (8) of the distinguishable features were derived from the gray level co-occurrence matrix. It was observed that some features such as GLSZM (glVariance) were statistically different between region 1 vs 3, 2 vs 3 and 2 vs 4, but not between region 1 vs 2, 1 vs 4 and 3 vs 4.

**Table 3:** List of features demonstrated statistically significant differences (p<0.05) between all regions.

Features group	Features
GLCM	Difference average
	Difference entropy
	Dissimilarity
	Inverse difference
	Inverse difference normalised
	Inverse difference moment
	Correlation
	Second measure of information correlation
GLRLM	Long runs emphasis
	Grey level nonuniformity
	Grey-level-nonuniformity-normalised
	Run percentage
	Run entropy
NGTDM	Complexity
	Strength

## 4. Discussion

The main purpose of this study was to assess the stability of PET radiomic features with varying reconstruction settings involving different configurations of synthetic lesions. From those features identified as stable, we determined the subset of features that can still demonstrate distinguishable and significant differences between image regions with varying radioactive heterogeneity. A phantom study was used to assess these properties and hence remove the complexities of physiologically induced confounding variables which may be introduced if the analysis is performed *in vivo*.

Four arrays of radioactivity filled syringes (7 in total to represent a synthetic tumour) were placed in the phantom and imaged for 80 min. Images were reconstructed with different reconstruction settings (TOF, Subsets, Iterations and FWHM Gusion filters). We extracted 78 radiomic features (GLCM, GLRLM, GLSZM, GLDZM and NGTDM), calculations were compliant with the Image Biomarker Standardization Initiative (IBSI). We calculated the COV for each feature with varying reconstruction parameters and categorized their COV values into 4 groups (stable,moderately stable, poorly stable, unstable). The results of this study indicated that different reconstruction settings have different influences on PET radiomic features. For instance, GLCM (Difference entropy, Inverse difference normalised), GLRL (Short runs emphasis, Run entropy), GLSZM (Zone size entropy), GLDZM (Zone distance entropy) were stable against all reconstruction settings, while GLRL (Long run low grey level emphasis) were unstable against most of reconstruction settings. NGTDM (Busyness) was moderately stable against subsets and unstable against filters.

The important role of TOF is measuring the variation in arrival time of the two emitted photons leading to localizing the emission point more precisely. TOF can improve the contrast and reduce the noise, and therefore a better signal to noise ratio. Interestingly in this study, the use of TOF had the lowest impact on radiomic features. The OSEM algorithm is an acceleration of the expectation maximization (EM) algorithm. However, a trade-off exists between the number of subsets and increasing noise and image quality. In addition, increasing number of iterations leads to increased noise (27). The number of iteration and subsets were found to have similar effects on all measured radiomic features. This may be due to the fact that OSEM reconstructions with n iterations and m subsets are equivalent to m iterations and n subsets and increasing either leads to an increased product of iterations (subset × iteration) which eventually leads to elevate the noise level. The largest variation of image features occurred with changing the FWHM of the gaussian filter. The role of smoothing utilizing the gaussian filter is to improve signal to noise. However, the spatial resolution will be reduced with larger FWHM which causes a more uniform intensity distribution and hence an impact on extracted texture feature variability.

This study differs from prior works in several ways such as many more features were extracted and hence reported than previous work. We also have an increased number of lesion configurations, heterogeneity activity levels and varied reconstruction parameters. As an example, (21), (22) and (23) extracted 27, 58 and 39 radiomic features respectively, while in our study, we extracted 78 radiomic features. Furthermore, Forgacs et al. utilised only 3 different numbers of iterations, 2 number of subsets and 2 FWHM Gaussian filters (21) whilst our study is based on 6 different levels of iterations, 5 levels of subsets and 8 FWHM Gaussian filter variations. Moreover, all radiomics features in this work were compliant with the Image Biomarker Standardization Initiative (IBSI). Hence, this study provides a more encompassing analysis of our knowledge of the robustness of features against different reconstruction settings whilst also exploring the utility of those features in distinguishing between heterogeneity activity distributions via Friedmans analysis.

The present findings seem to be consistent with other research which found that varying reconstruction settings has variable influence on the stability of different PET radiomic features. As an example, Gallivanone et al. assessed the impact of different reconstruction settings (i.e. filters, iterations and subsets) on different radiomic features (22). Their results found that subsets and matrix size had lowest and greatest impact on the stability of features, respectively. In our study, in comparison to Gallivanone et al., about 19, 22, 17 features (from 36 common features) had the same COVs against subsets, iterations and filter size, respectively. Our results confirm Gallivanone et al.’s finding that dissimilarity (GLCM), Short run emphasis (GLRLM), Small zone emphasis (GLSZM), strength (NGTDM) has high stability. Low gray-level run emphasis and Long run low gray-level emphasis (GLRLM), Large zone low gray-level emphasis (GLSZM) are unstable.

Doumou et al. studied the impact of image smoothing, segmentation and quantisation on the stability of 57 heterogeneity features (28). For the 38 features in common with our study, 12 features had good agreement in the effect of FWHM Gaussian filter. As an example, Inverse difference normalised (GLCM) and strength (NGTDM) were stable and small zone low emphasis and large zone low emphasis (GLSZM) were unstable against varying Gaussian filter size in both studies.

In a study by Shiri et al., 100 radiomic features were extracted from patient and phantom images with different reconstruction settings (14). Our results are consistent with their findings, in that the Short run emphasis (GLRLM), zone percentage (GLSZM), correlation and Inverse difference moment (GLCM) have small variability against subsets and FWHM filters. In Shiri at al., four different reconstruction algorithms (OSEM, OSEM+PSF, OSEM+TOF and OSEM+PSF+TOF) were included, but in our study we only included two reconstruction algorithms in order to assess the impact of TOF, specifically OSEM with and without TOF.

In another study, Forgacs et al. used inhomogeneous tumor insert (7 syringes) placed in a cylindrical phantom and imaged with different acquisition times and reconstruction settings (21). According to their strategy, reliable heterogeneity parameters must be volume independent, reproducible, and appropriate for detecting heterogeneity levels. Entropy, Correlation, Homogeneity and Contrast were found to have low variation with varying acquisition times and reconstruction settings (21). In our study, 3 out of these 4 features were found to have very low COV when varying all of the tested reconstruction settings.

Bailly et al. assessed the robustness of 15 features with matrix size, number of iterations, Gaussian post-filtering, noise and the reconstruction algorithm (29). For the 13 features in common with our own study, 38% and 54% of them showed the same COVs in number of iterations and FWHM Gaussian filter, respectively.

There are several causes for the differences between our results and previous work in this area. Firstly, the statistical methods used to analyze the results are unique. Second, the range of categorizations differ from one study to another. For instance, we categorized the features into 4 groups based on the COV values, but in the Bailly study, they used only 3 categorizations. Furthermore, other factors such as segmentation methods, bin size and default reconstruction settings may have a considerable difference between studies.

In this study, we performed further statistical analysis to determine the ability of what we have defined as stable features in distinguishing between phantom inserts with different heterogeneity. The Friedman test (non-parametric) was used for these purposes on 43 (out of 78) features. Thirty five features were excluded in this analysis due to their instability against reconstruction settings. The Friedman test was performed for each combination (shape1 vs shape2, shape1 vs shape3, shape1 vs shape4, shape2 vs shape3, shape2 vs shape4 and shape3 vs shape4) of heterogeneity configurations to determine whether or not there was a statistically significant power in each texture feature distinguishing between image regions of the varying insert configurations when using different reconstruction parameters. This study found that 15 features demonstrated statistically significant differences (p<0.05) between all regions. Therefore, these 15 features may be reasonably considered as stable and capable of discerning, with statistical significance, differences between phantom objects with varying heterogeneity. This has, to the best of the authors’ knowledge, not previously been presented before in the PET radiomics literature.

The study has some limitations. First, the impact of interpolation, segmentation and quantization have not taken into account. Whypra et al. (18) , Leijenaar et al. (13) and Lu et al. (30) have investigated the effect of these parameters. We used a fixed isotropic voxel dimension, delineation and bin size with all of reconstructed images to minimize the impact of these parameters. This was also recommended by the IBSI (31). Second, this study like others was carried out in static conditions and did not include any radio kinetic component as we aimed to report on the stability of PET radiomic features against different PET reconstruction parameters. Third, this study is concerned with the stability of radiomic features for different image reconstruction parameters. An image phantom is used for these purposes; test-retest repeatability would measure variations in phantom filling rather than variability introduced by the reconstruction alogrithm. Future work will explore if these findings are consistent across different reconstruction algorithms provided by different manufacturers. Fourth, this study did not involve clinical data. However, the phantom study informs the variabilities that may exist in a clinical context. A similar study using clinical data may be conducted using the methods used in this study. This may serve as a future work.

## 5. Conclusions

The purpose of this work was to determine stable PET radiomic features that do not vary with changing PET reconstruction parameters but maintain the ability to distinguish between different synthetic tumor inserts with varying heterogeneity. Our study showed that forty three (55%) features were found to be stable against reconstruction settings. Fifteen features were found to have an ability to capture heterogeneous differences between lesions. These features are: (1) stable to reconstruction parameters and (2) capable of providing statistically significant differences in the presence of different levels of phantom designed spatial heterogeneity. Further research involving clinical data using a similar approach could contribute to a deeper understanding of the clinical application and translation of radiomic features.

## Data Availability

The raw data supporting the conclusions of this article will be made available by the authors upon request. Requests to access these datasets should be directed to Emad Alsyed, alsyed@kau.edu.sa.
